# Prognostic Value of IGF2 mRNA-Binding Protein 3 (IGF2BP3) Intratumoral Expression in Melanoma Patients at the Time of Diagnosis: Comparative Analysis of RT-qPCR Versus Immunohistochemistry

**DOI:** 10.3390/cancers14092319

**Published:** 2022-05-07

**Authors:** Beatriz Sánchez-Sendra, Silvia Pérez-Debén, José F. González-Muñoz, Amelia Murgui, Carlos Monteagudo

**Affiliations:** 1 Department of Pathology, University of Valencia, 46010 Valencia, Spain; sansenbe@uv.es; 2 Oncology Area, Skin Cancer Research Group, Biomedical Research Institute INCLIVA, 46010 Valencia, Spain; sperez@incliva.es (S.P.-D.); jgonzalez@incliva.es (J.F.G.-M.); 3 Department of Biochemistry and Molecular Biology, University of Valencia, 46100 Valencia, Spain; maria.murgui@uv.es; 4 Department of Pathology, University Clinic Hospital of Valencia, 46010 Valencia, Spain

**Keywords:** melanoma, IGF2BP3, RT-qPCR, IHC, biomarkers, distant metastasis

## Abstract

**Simple Summary:**

Around 80% of skin cancer deaths are due to melanoma. An accurate prognosis of melanoma clinical behavior from primary tumors is important for therapeutic patient management, currently based on histopathological features. The aim of our retrospective study was to investigate the clinical significance of IGF2BP3 mRNA and protein expression in melanoma progression and to evaluate which quantification method, RT-qPCR or immunohistochemistry, provides a more reliable prognostic value of IGF2BP3 expression in primary tumors. We found that IGF2BP3 mRNA expression correlated better with clinicopathologic melanoma features than the corresponding proteins and that patients with higher IGF2BP3 mRNA levels were at more risk for earlier development of metastasis, confirming its impact on melanoma survival. Our findings support the use of IGF2BP3 mRNA levels as an independent prognostic biomarker and the implementation of its RT-qPCR analysis for routine melanoma assessment, even for the earliest stages, to improve melanoma clinical outcomes and individualized treatment.

**Abstract:**

Screening for prognostic biomarkers is crucial for clinical melanoma management. Insulin-like growth factor-II mRNA-binding protein 3 (IGF2BP3) has emerged as a potential melanoma diagnostic and prognostic biomarker. It is commonly tested by immunohistochemistry (IHC). Our study retrospectively examines IGF2BP3 mRNA and protein expression in primary melanomas, their correlation with clinicopathologic factors, clinical outcome, and selected miRNAs expression, and their efficiency in predicting melanoma progression and survival. RT-qPCR and IHC on IGF2BP3 expression were performed in 61 cryopreserved and 63 formalin-fixed paraffin-embedded primary melanomas, respectively, and correlated to clinicopathologic factors, distant metastasis-free survival (DMFS), and melanoma -specific survival (MSS). The correlation between RT-qPCR and IHC was significant but moderate. IGF2BP3 mRNA showed a stronger association with clinicopathologic factors (Breslow thickness, ulceration, mitosis rate, growth phase, development of metastasis, and melanoma-specific survival) than its protein counterpart. Interestingly, higher IGF2BP3 mRNA expression was detected in primary melanomas that further metastasized to distant sites and was an independent prognostic factor for the risk of unfavorable DMFS and MSS. RT-qPCR outperformed IHC in sensitivity and in predicting worse clinical outcomes. Therefore, RT-qPCR may successfully be implemented for routine IGF2BP3 assessing for the selection of melanoma patients with a higher risk of developing distant metastasis and dying of melanoma.

## 1. Introduction

Cutaneous malignant melanoma accounts for around 80% of all skin cancer-related deaths worldwide due its great potential to metastasize [1]. The surgical resection of primary melanomas at the earliest stages of development is currently an effective treatment associated with prolonged disease-free survival. Moreover, the implementation of new modalities of therapies, particularly combination immunotherapy, has increased the survival of patients with advanced melanoma from a 2-year survival of 15% to a 5-year survival of 50% [2]. However, a subset of patients (15–25%) with resectable melanoma develop metastases and die from melanoma [3].

The ability to predict metastatic dissemination from primary cutaneous melanoma is a decisive factor for the clinical outcome in patients with melanoma and for the choice of better strategies for patient management. The standard clinical and histopathological features, such as primary tumor thickness, the presence of ulceration, lymph nodes metastases, and distant metastases, included in the staging system for cutaneous melanoma of the American Joint Committee on Cancer (AJCC) are considered the main prognostic determinants of melanoma [4]. However, these factors still fail in accurately predicting clinical outcomes in a significant number of melanoma cases [3].

Current research is focused on searching for molecular markers expressed in the primary tumor that help to more accurately predict the clinical behavior and prognosis of melanomas. On the one hand, the deregulation of some microRNAs (miRNAs) has been described to influence the development, pathogenesis, invasiveness, and progression of melanoma and has been proposed as a strong prognostic factor for melanoma [3,5,6,7,8,9]. For this reason, the present work includes an analysis of selected miRNA expression. Other already known molecular markers in the course of melanoma are those related to the gene mutations (the BRAF, NRAS, KIT, NF1, and TERT promoters, as well as the CDKN2A and PTEN deletions) [10]. On the other hand, insulin-like growth factor-II mRNA-binding protein 3 (IGF2BP3) has also been recently highlighted to be a noteworthy marker of melanoma diagnosis and prognosis [11,12,13,14]. In fact, the depletion of CDR1as circular RNA promotes invasion and metastasis through an IGF2BP3-mediated mechanism [15].

IGF2BP3 is a fetal oncoprotein not expressed in normal adult tissues [16]. However, increased levels of IGF2BP3 have been identified in many cancers including adenocarcinomas of the pancreas, kidney, lung, breast, esophagus, cervix, endometrium, and cutaneous melanomas [17]. Moreover, IGF2BP3 played significant roles in cell proliferation, migration, and adhesion during cancer progression [18,19], and it has been recognized as an indicator for cancer progression and metastasis and a predictor of poor prognosis for many types of cancers [17,20,21,22].

Regarding melanoma, IGF2BP3 was found to be expressed in malignant melanomas but not in most benign nevi, even when dysplastic features were present. IGF2BP3 immunostaining was higher in metastatic melanomas than in thin primary melanomas [14,23,24]. Moreover, patients with a tumor thickness lower than 4.0 mm and positive IGF2BP3 expression had a significantly worse melanoma-specific survival than those without IGF2BP3 expression [11,12].

To date, most IGF2BP3 studies on melanoma have analyzed it by immunohistochemistry (IHC). IHC is the main supplementary tool in the histopathological evaluation of many tumor markers, but it often provokes discussion as to its reproducibility and its inability to provide quantitative data. This method carries significant intra- and inter-observer variability and is not accurate enough when it comes to quantification [24]. In contrast, other more sensitive, unbiased, reproducible, and accurate quantification methods, such as reverse transcription quantitative real time polymerase chain reaction (RT-qPCR), are being increasingly evaluated in order to achieve a more reliable prognostic value of tumor markers. In this study we retrospectively analyzed the gene and protein expression of IGF2BP3 in primary cutaneous melanomas, by RT-qPCR and IHC, respectively, and compared them with clinical and histopathological variables and with prognostic parameters. Our goal was to investigate the clinical significance of IGF2BP3 (mRNA and protein) in melanoma progression and evaluate which quantification method, RT-qPCR or IHC, provides a higher prognostic value of the expression of IGF2BP3 among primary melanomas.

## 2. Materials and Methods

### 2.1. Human Melanoma Tissues

Melanoma primary tumors from a total of 70 patients with cutaneous melanoma were included in this study. The samples comprised superficial spreading melanoma (SSM) (*n* = 52, 74.29%), nodular melanoma (NM) (*n* = 8, 11.43%), lentigo maligna melanoma (LMM) (*n* = 5, 7.14%), and acral lentiginous melanoma (ALM) (*n* = 5, 7.14%) (Table 1). All tumor specimens were received at the Department of Anatomic Pathology, Hospital Clínico Universitario, Valencia, Spain, from July 2003 to December 2014. The medical records from all patients were reviewed and clinical follow-up locked in November 2020.

Primary melanomas were collected by manual macrodissection to ensure maximum tumor tissue content. In most cases (*n* = 54), biopsies were split into two parts. A tumor slice immediately adjacent to the thickest area of the tumor was selected for RNA extraction and was immediately frozen in liquid nitrogen and stored at −80 °C, and the remaining fresh tumor tissue was formalin-fixed and paraffin-embedded (FFPE) for routine diagnosis. Nine biopsies were only formalin-fixed and paraffin-embedded, and another seven biopsies were only cryopreserved. Therefore, a total of 63 samples were analyzed by IHC and 61 samples by RT-qPCR.

The following clinical variables were considered: Breslow thickness (stratified by ≤1, 1.01–2, 2.01–4, or >4 mm), ulceration (absent versus present), growth phase (radial versus vertical), mitotic index (number of mitosis/mm^2^), metastasis (absent versus present), metastasis stage along the clinical follow-up (in-transit, lymph node, and distant metastasis), and histological type (SSM, NM, LMM, and ALM) (Table 1). Clinical follow-up, with particular emphasis on the development of distant metastases and melanoma mortality, ranged from 6.6 to 210.4 months (mean 96.1, median 100.9 months). The survival rates for 70 patients with different melanoma progression stages were calculated. Melanoma-specific survival (MSS) was defined as the period from the initial melanoma diagnosis to the date of the last follow-up or death from melanoma (event). Patients who were alive at last follow-up or who died without evidence of melanoma were considered censored. Distant metastasis-free survival (DMFS) was defined as the length of time from initial melanoma diagnosis to the date of distant metastasis occurrence.

Ethics approval and consent to participate: this study was approved and supervised by the Ethical and Scientific Committees of the Hospital Clínico Universitario of Valencia (Prometeo II/2015/009), and all research was performed in accordance with the relevant guidelines and regulations. All melanoma patients enrolled in this study provided written informed consent.

### 2.2. IGF2BP3 Immunohistochemical Analysis

Immunohistochemical analysis was performed on 5-μm formalin-fixed and paraffin-embedded sections from tissue microarray (TMA) blocks composed of primary tumor cores (2 mm diameter) punched from representative tumor areas with at least two representative duplicate cores for each case. The immunohistochemical staining was carried out using two different antibodies from different manufacturers, a rabbit monoclonal antibody against IMP-3, an alias for IGF2BP3 (ab179807,1:75; Abcam, Cambridge, UK), and a mouse monoclonal antibody against IMP-3 from Dako (M3626, clone 69.1, 1:100; Agilent, CA, USA). Both monoclonal antibodies (MAbs) were selected because they are reported to give excellent results in FFPE human tissue samples. Clone 69.1 (Dako/Agilent) is a mouse MAb obtained using amino acid 2–580 of the Escherichia coli recombinant protein as immunogen (the precise epitope is unknown) by the hybridoma technology. Clone EPR12021-114 (Abcam) is a rabbit MAb obtained using recombinant fragment aa 1–200 (the exact sequence, however, is proprietary) as immunogen by the hybridoma technology. Tissue TMA sections were deparaffinized and antigen retrieval performed at 98 °C for 20 min in high-pH buffer using Dako PT link. Slides were rinsed with TBS and endogenous peroxidase activity blocked with Dako peroxidase blocking solution for 10 min. After blocking, the sections were incubated with the primary IGF2BP3 antibody diluted in Dako antibody diluent solution at room temperature for an hour. The sections were then incubated for 30 min with Envision FLEX HRP. Staining was developed with Dako DAB-containing EnVision FLEX substrate buffer for 10 min. Then, the slides were rinsed with distilled water and counterstained with hematoxylin followed by a running-tap water rinse and mounting. Cytoplasmic staining was regarded as positive for IGF2BP3 expression. IGF2BP3 protein expression quantification was performed according to the H-score, based on multiplying the staining intensity [negative (0), weak (1), moderate (2), or strong (3)] by the percentage of IGF2BP3-positive cells (0–100%). IGF2BP3 immunostaining reactions were evaluated by an expert pathologist (CM) with no knowledge of the clinical or molecular data. H-score 1 values correspond to the ones obtained with Abcam antibody and H-score 2 to Dako antibody.

### 2.3. RNA Extraction

For RNA extraction, cryopreserved primary melanoma tissues were first disaggregated with a pre-chilled scalpel into tiny portions and mechanically homogenized in 600 μL of Lysis/Binding buffer. Then, the total RNA was extracted using the mirVana miRNA isolation kit (Ambion, Austin, TX, USA) according to the manufacturer’s instructions. RNA concentration and quality were determined on a NanoDrop One Spectrophotometer (Thermo Fisher Scientific, Waltham, MA, USA) and stored at −80 °C.

### 2.4. IGF2BP3 mRNA Quantification by RT-qPCR

The relative quantification of IGF2BP3 mRNA was also performed from the same primary melanoma tissues by RT-qPCR. RT was performed using the High Capacity cDNA Reverse Transcription Kit and adding an Rnase inhibitor (Lifetechnologies, Carlsband, CA, USA). In 25-μL reactions, 75 ng of total RNA was converted to cDNA. For RT-qPCR analysis, 1 μL of cDNA was used in 10-μL qPCR reactions by adding TaqMan^®^Gene Expression Master Mix and TaqMan Gene Expression Assays for the target gene IGF2BP3 (assay ID: Hs00559907_g1) and the endogenous reference gene 18S (assay ID: Hs99999901_s1). 18S ribosomal RNA (rRNA) is the structural RNA for the small component of eukaryotic cytoplasmic ribosomes and thus one of the basic components of all eukaryotic cells. This is one of the reasons why 18S is one of the most commonly used genes as an endogenous reference/normalizer for gene expression studies. 18S expression is usually high and stable in most cell types. Another abundant referent gene is GAPDH, but it was reported that its expression is deregulated during melanoma progression [25], which makes it unsuitable to be used as an appropriate housekeeping gene for mRNA expression analysis in melanoma patients, and, in contrast, it was proved that the expression of 18S is not modified during the different stages of melanoma progression [26]. Because of this, it was the gene we selected to be used as the endogenous reference for mRNA qPCR analysis.

All reactions were performed in triplicate in 384-well plates on a 7900 HT Fast Real Time PCR system (Lifetechnologies, Carlsband, CA, USA). Both RT and qPCR negative controls were included for each assay. For the relative quantification of mRNA expression, the 2^-ΔCt^ method was used, and the results were analyzed using Expression Suite software (Lifetechnologies, Carlsband, CA, USA).

### 2.5. miRNAs Quantification by RT-qPCR

From human melanoma samples, the relative quantification of 10 mature intratumoral miRNAs previously described in melanoma (hsa-miR-125b-5p, hsa-miR-137, hsa-miR-182-5p, hsa-miR-191-5p, hsa-miR200c-3p, hsa-miR-205-5p, hsa-miR-21-5p, hsa-miR-211-5p, hsa-miR-221-3p, hsa-miR-9-5p) (Sánchez-Sendra B., et al. 2018) was carried out by reverse transcription quantitative real time polymerase chain reaction (RT-qPCR). Reverse transcription (RT) was performed using a TaqMan MicroRNA Reverse Transcription Kit using an RNase inhibitor (Lifetechnologies, Carlsband, CA, USA). In 45-μL reactions, 200 ng of total RNA was converted to cDNA. RT reactions were multiplexed by customizing the RT primer pool with miRNA-specific RT primers of interest, following the manufacturer’s recommendations. The TaqMan MicroRNA Assays used (Lifetechnologies, Carlsband, CA, USA) are listed in Table 2. RT-qPCR was performed with 1 μL of cDNA in 10-μL qPCR reactions by using TaqMan Universal Master Mix II, no UNG and TaqMan^®^MicroRNA Assays for target miRNAs (Lifetechnologies, Carlsband, CA, USA). Small nuclear RNU48 was used as the endogenous reference gene. For PCR assays, the number of replicates, the negative controls, and the relative quantification method were the same of those mentioned in the above paragraph. See the miRNAs expression analysis results in the Supplementary Materials (Figures S1 and S2, Tables S1 and S2).

### 2.6. Statistical Analysis

Statistical analysis was performed using GraphPad Prism V.6.01 (GraphPad Prism Software, Inc., San Diego, CA, USA) and R software [27]. The following R packages were used: Stats version 3.6.3 for the Wilcoxon rank test, Kruskal–Wallis rank test, and logistic regression models; psych version 2.0.9 for the Spearman correlation coefficient and test; and Survival version 3.2–7 for survival analysis (Kaplan–Meier curves and the Cox proportional hazard model).

The Wilcoxon rank sum test was used to analyze the association between IGF2BP3 (mRNA and protein) expression and the two-categorical clinicopathologic and prognostic parameters and also to analyze the association between miRNA expression and IGF2BP3 (mRNA and protein) expression treated as a two-categorical variable (above or below IGF2BP3 median value), while the Kruskal-Wallis rank sum test was used to compare categorical parameters defined by more than two different groups. The Spearman correlation coefficient r was used as a measure of the strength and direction of the relationship between IGF2BP3 mRNA and protein expression and continuous clinicopathologic variables such as Breslow thickness, mitotic index, and miRNA expression. The Spearman correlation was also used to analyze the correlation between both IGF2BP3 mRNA and IGF2BP3 protein levels, treated as continuous variables.

Survival probabilities associated with melanoma-specific survival (MSS) and with distant metastasis-free survival (DMFS) were calculated using the Kaplan-Meier method and were examined by a log-rank test. The influence of each variable on survival (MSS and DMFS) was assessed using univariate and multivariate Cox proportional hazard models. Breslow thickness, ulceration, and IGF2BP3 (mRNA or protein) expression were the variables included in the Cox models. These models were constructed to identify the independent prognostic factors of clinical outcome, considering hazard ratios (HR), 95% confidence interval (CI), and *p*-values. Multivariate analysis taking into account the same three covariables was also performed to determine the independent prognostic factors of distant metastasis by a logistic regression model. All statistics were considered to be statistically significant when the *p*-value was less of 0.05.

## 3. Results

### 3.1. IGF2BP3 mRNA and Protein Follow the Same Expression Pattern and Correlate with Conventional Clinicopathologic Prognostic Factors in Human Primary Melanomas

To decipher the prognostic value of IGF2BP3 for melanoma progression, we analyzed the IGF2BP3 expression in primary melanomas, measured by RT-qPCR and immunohistochemistry, and studied its potential correlation with widely accepted clinicopathologic prognostic factors. The clinicopathologic features of the primary tumors included in our study are summarized in Table 1.

**Figure 1:**
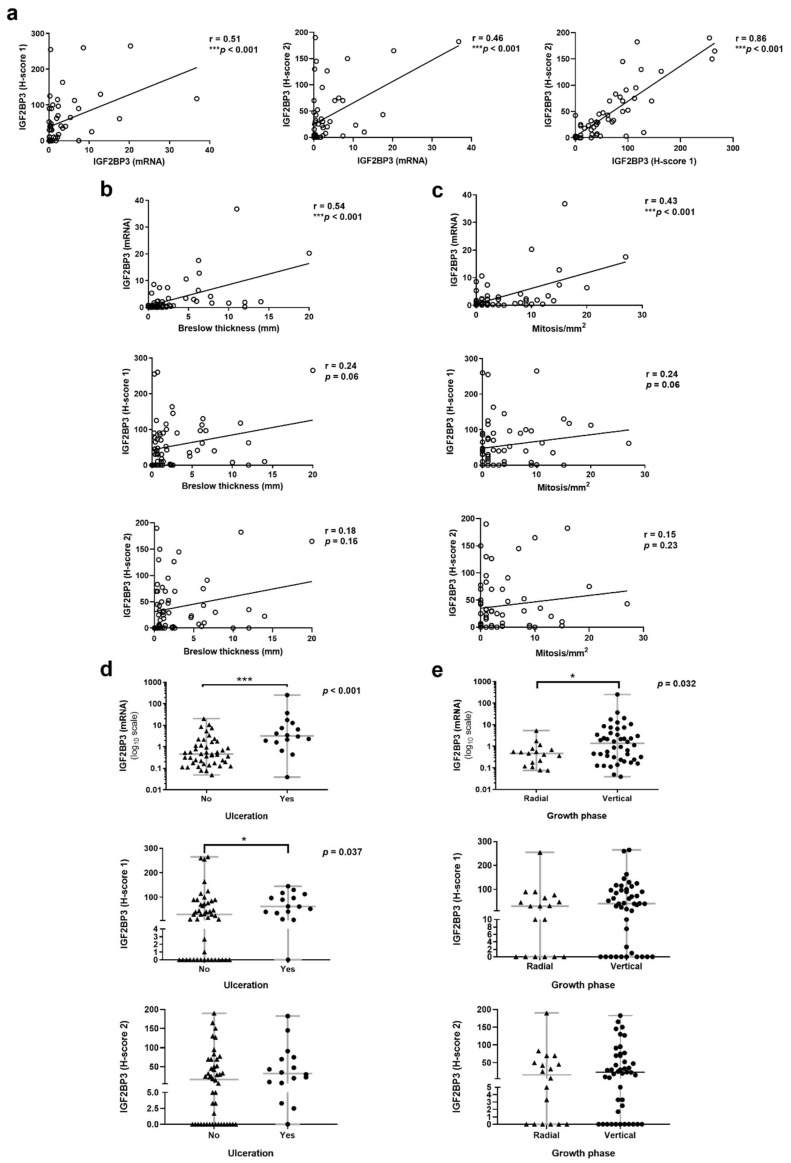
Correlation between IGF2BP3 expression and clinicopathologic features in primary melanomas. (**a**) Correlation between IGF2BP3 mRNA expression and its counterpart IGF2BP3 protein (H-score 1 and H-score 2), and the correlation between both H-scores. (**b**) Correlation of IGF2BP3 mRNA and protein (H-score 1 and 2) according to Breslow thickness, (**c**) the number of mitoses per mm^2^, (**d**) ulceration status, and (**e**) growth phase of the primary melanomas. * *p* < 0.05 and *** *p* < 0.001. For all the point plots graphed in this article, the horizontal gray line represents the median value (second quartile or Q2), the lowest point of lower whiskers is the minimum value of the variable, and the highest point of the upper whiskers is the maximum value of the variable.

First, we analyzed if a correlation between the mRNA (RT-qPCR) and protein (IHC) expression values of IGF2BP3 exists in the primary melanoma tissue samples, where both the IGF2BP3 gene and protein were measured (*n* = 54). The correlation analysis showed a significant positive but moderate correlation between IGF2BP3 mRNA and its protein counterpart value for the two antibodies used in this study (Spearman correlation coefficient: 0.51, *p* < 0.001 and 0.46, *p* < 0.001 for H-score 1 and H-score 2, respectively), as shown in Figure 1a. Moreover, both H-scores showed a considerably significant positive correlation between them (Spearman correlation coefficient: 0.86, *p* < 0.001) (Figure 1a).

**Figure 2:**
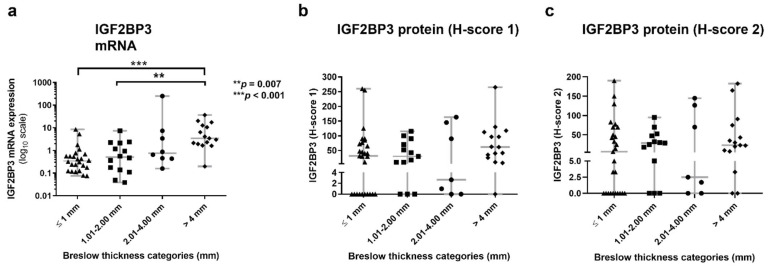
Association of IGF2BP3 (**a**) mRNA expression, (**b**) protein levels measured according to H-score 1, and (**c**) protein levels according to H-score 2 with Breslow thickness categories. ** *p* < 0.01 and *** *p* < 0.001.

Overall, IGF2BP3 mRNA correlates with more clinicopathologic factors and with more significance than its counterpart protein. Particularly, we found a significant and positive correlation between IGF2BP3 mRNA expression and both Breslow thickness and mitotic index in primary melanomas (Table 3) (Figure 1b,c). Regarding the IGF2BP3 protein (H-score 1 and H-score 2), although correlation trends with Breslow thickness and mitotic index were found, they were not significant (Table 3) (Figure 1b,c). Similarly, when Breslow thickness was stratified into four categories, both mRNA and protein (only H-score 1) showed the higher value at the poor prognosis stage (T4), but there were only significant differences among the Breslow stages for IGF2BP3 mRNA (*p* < 0.001) (Table 3), specifically in the pairwise comparisons stage T4 versus T1 and stage T4 versus T2 (*p* = 0.0001 and *p* = 0.0073, respectively) (Figure 2).

Furthermore, ulcerated primary melanomas and those with vertical growth phase exhibited higher levels of IGF2BP3 mRNA and protein (H-score 1 and H-score 2) than those not ulcerated and/or with radial growth, this being significant in the case of IGF2BP3 mRNA expression. IGF2BP3 protein (H-score 1) levels were significantly higher only in the presence of ulceration, not in vertical growth phase. No significant differences were observed when studying the IGF2BP3 protein with the Dako antibody (H-score 2) (Table 3) (Figure 1d,e).

### 3.2. IGF2BP3 Expression in Primary Melanoma Tumors Is Associated with Metastasis Development and Progression and with Melanoma-Specific Survival

**Figure 3:**
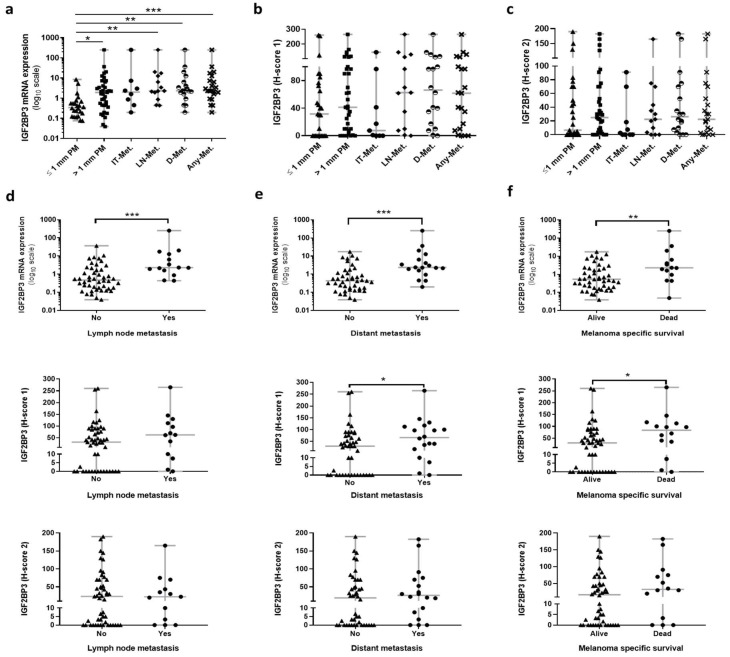
Association of IGF2BP3 mRNA and protein expression (H-score 1 and H-score 2) in primary melanomas according to clinical behavior. Comparison between the relative expression of (**a**) IGF2BP3 mRNA, (**b**) IGF2BP3 protein levels (H-score 1), and (**c**) IGF2BP3 protein levels (H-score 2) along the different steps of melanoma progression: thin primary melanomas (≤1 mm PM), thick primary melanomas (>1 mm PM), in-transit metastases (IT-Met), lymph node metastases (LN-Met), distant metastases (D-Met), and in any metastases (Any-Met). Association of IGF2BP3 mRNA and protein (H-scores 1 and 2) expression in primary tumors based on the further development of (**d**) lymph node metastasis, (**e**) distant metastasis, and (**f**) melanoma-specific death. * *p* < 0.05, ** *p* < 0.01, and *** *p* < 0.001.

A progressive increase in IGF2BP3 mRNA expression was found along the full spectrum of melanoma progression, from thin primary melanomas to distant metastatic tumors (*p* < 0.001). Precisely, significant differences were detected when thin melanomas (≤1 mm) were compared with thick ones (>1 mm) and with the development of lymph node metastasis, distant metastasis, or any metastasis in general (*p* = 0.019, *p* = 0.002, *p* = 0.001, and *p* = 0.0003, respectively). This tendency was not observed for the IGF2BP3 protein (H-score 1 and H-score 2) (Figure 3a–c).

Of the primary melanomas, 27.1% (19/70) further progressed to distant metastatic tumors, and 73.7% (14/19) of these patients died of melanoma (Table 1). IGF2BP3 mRNA was significantly higher in primary tumors that metastasized to the nearest lymph node and to distant sites (*p* < 0.001 and *p* < 0.001, respectively) (Figure 3d,e). The IGF2BP3 protein (H-score 1) also showed this behavior but was only significantly higher for primary tumors that progressed to distant metastasis (*p* = 0.031) (Figure 3d,e) (Table 3). This tendency was also observed in the primary tumors of patients who died from melanoma for both IGF2BP3 mRNA and protein (H-score 1) (*p* = 0.009 and *p* = 0.029, respectively) (Figure 3f) (Table 3). The IGF2BP3 protein (H-score 2) also showed this tendency but only for distant metastasis and melanoma specific survival and without reaching statistical significance (Figure 3e,f).

### 3.3. IGF2BP3 mRNA Has a Prognostic Value for DMFS and MSS but Does Not Predict Distant Metastasis Onset

**Figure 4:**
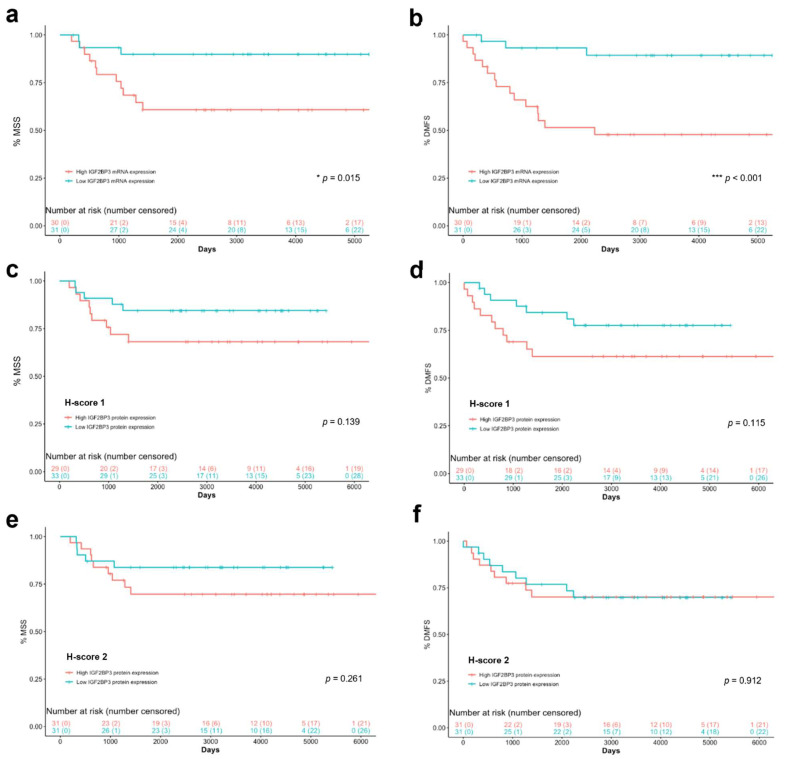
Kaplan–Meier curves for MSS and DMFS of melanoma patients depending on IGF2BP3 mRNA and IGF2BP3 protein (H-scores 1 and 2) expression being down (blue) or over (red) their corresponding median expression values. Influence of IGF2BP3 mRNA expression affecting the (**a**) MSS and (**b**) DMFS of patients, respectively, and the influence of the IGF2BP3 protein H-score 1 on (**c**) MSS and (**d**) DMFS and of the IGF2BP3 protein H-score 2 on (**e**) MSS and (**f**) DMFS. * *p* < 0.05 and *** *p* < 0.001.

In survival analysis, the population was dichotomized into patients with high or low IGF2BP3 expression using the median values for IGF2BP3 mRNA or protein expression. The Kaplan–Meier survival analysis showed that IGF2BP3 mRNA expression was significantly associated with clinical endpoints such as distant metastasis-free survival (DMFS) and melanoma-specific survival (MSS). Both (DMFS and MSS) were significantly shorter in patients with primary melanomas showing IGF2BP3 mRNA expression levels above the median (median MSS: 60.81% vs. 89.88%, *p* = 0.015 and median DMFS: 47.79% vs. 89.33%, *p* < 0.001, respectively) (Figure 4a,b). The same tendency was observed for IGF2BP3 protein levels (H-score 1 and H-score 2) but without reaching the statistical significance (Figure 4c–f).

To determine if IGF2BP3 (mRNA and protein) expression could improve the prognosis value of the two main clinicopathologic variables (Breslow thickness and ulceration), we performed Cox univariate and multivariate analyses for both DMFS and MSS (Table 4). In the univariate Cox model, IGF2BP3 mRNA expression was a significant predictive factor for both DMFS and MSS, and, even when IGF2BP3 mRNA expression was included in the multivariate predictive Cox model, all three considered variables (Breslow thickness, ulceration, and IGF2BP3 mRNA expression) were predictive of poor outcomes (Table 4). Then, IGF2BP3 mRNA expression was proved to be an independent prognostic factor for DMFS (HR, 1.016; 95% CI: 1.005–1.028; *p* = 0.004) and MSS (HR, 1.024; 95% CI: 1.001–1.048; *p* = 0.044) together with thicker and ulcerated melanomas. Furthermore, as the IGF2BP3 mRNA hazard ratio indicates, the higher the IGF2BP3 mRNA expression level, the higher the risk of distant metastasis development and, therefore, of shorter patient survival (Table 4). However, Breslow was the best independent predictor of both DMFS and MSS. On the contrary, IGF2BP3 protein expression (H-scores 1 and 2) was not individually significant in the univariate analysis for DMFS (*p* = 0.078 and *p* = 0.487 for H-score 1 and 2, respectively) nor for MSS (*p* = 0.091 and *p* = 0.258 for H-score 1 and 2, respectively) and was not independent of Breslow and ulceration for DMFS (p=0.053 and *p* = 0.651 for H-score 1 and 2, respectively) nor for MSS (*p* = 0.322 and *p* = 0.678 for H-score 1 and 2, respectively) in the multivariate model.

In the multivariate logistic regression analysis, neither IGF2BP3 mRNA expression (*p* = 0.485) nor protein expression (*p* = 0.508 for H-score 1, and *p* = 0.545 for H-score 2) were predictive factors for distant metastasis onset. Only Breslow thickness turned out to be a significant predictor factor for distant metastasis development (*p* = 0.011 for the model with IGF2BP3 mRNA expression, *p* = 0.014 for the model with IGF2BP3 H-score 1 protein expression, and *p* = 0.016 for H-score 2) (Table 5).

## 4. Discussion

Cutaneous melanoma is considered the skin cancer with the highest mortality rate (55,000 cases/year). Furthermore, the 5-year survival rate for patients with resectable melanomas developing metastasis is low (14%) [28]. Defining an accurate prognosis for patients with primary melanoma is an essential clinical goal. Currently, patient management is based mainly on the histopathological features. However, patients with similar clinicopathologic primary melanomas can present different clinical outcomes. For this reason, besides histopathological characteristics, screening for reliable prognostic biomarkers is mandatory for the therapeutic management of cutaneous melanoma. One major obstacle is the lack of consensus on reproducible methodology paving the way for comparable results and clinical implications.

In this study, we evaluated the prognostic performance of two methods for assessing IGF2BP3 gene expression, one based on protein quantification (IHC) and the other on mRNA quantification (RT-qPCR) in the same cohort of melanoma patients. Indeed, in order to obtain more reliable results, the IGF2BP3 protein was analyzed using two different antibodies. From a cytophysiological perspective, and as central functional units in many complex biological pathways, proteins are a subject of much interest in various areas of translational medicine, including diagnostic biomarker discovery, drug discovery, and personalized medicine. However, measuring global protein levels directly in human tissue samples has traditionally presented many challenges, as well as major difficulties including reproducibility [29]. Indeed, mRNA analysis is gaining control in providing diagnostic and prognostic information. Many cancer screens utilize mRNA analysis to detect, characterize, and predict the risk of disease [30]. Examples of these are the microarray-based clinical tests, such as MammaPrint (Agendia) [31] and ColoPrint (Agendia) [32] for breast and colon cancer recurrence risk prognosis, respectively, and the rising RNA seq-based tests, which are having significant prognostic and therapeutic relevance to cancer due to their capability of high reproducibility, accuracy, and precision [30,33,34]. Measuring mRNA expression levels is cheaper but is insufficient to determine protein levels because correlations between mRNA expression and protein abundance are relatively low [35,36,37]. Correlations between mRNA and protein levels vary greatly among genes depending on regulatory processes that govern the rates of transcription, translation, posttranscriptional and posttranslational modifications, and protein/mRNA degradation. They also vary between experiments, organisms, and applied methodologies [38,39]. However, mRNA expression and protein abundance were highly correlated in cancer biomarkers and drug targets, implying that mRNA expression levels may, in some cases, be sufficient to determine protein abundance [39]. In this context, the present work analyzes and compares the prognostic value of IGF2BP3 mRNA versus protein expression in primary melanomas in terms of their efficiency to predict the clinical outcomes of patients. IGF2BP3 is a potential melanoma diagnostic and prognostic biomarker, and, in our study, IGF2BP3 mRNA and its protein counterparts (H-scores 1 and 2) showed a significant and moderate positive correlation. This approach would allow for easier estimates of protein levels from mRNA measurements.

To the best of our knowledge, this is the first melanoma study that directly compares two different IGF2BP3 quantification methods, IHC versus RT-qPCR, regarding their ability to predict melanoma progression and survival. There are no published data about IGF2BP3 intratumoral detection by RT-qPCR in melanoma. Most melanoma and other malignancies studies analyze this marker by immunodetection in formalin-fixed paraffin-embedded tumor samples [11,12,14,17,23]. Nevertheless, IGF2BP3 was analyzed by RT-qPCR in intrahepatic cholangiocarcinoma (ICC) to validate the results previously observed using IHC [40]. In this study, both IGF2BP3 mRNA and protein followed the same trend, exclusively detected in intrahepatic tumors but not in normal-appearing liver away from ICC. However, there was no significant correlation between the IGF2BP3 protein expression level and the mRNA level detected by RT-qPCR [28]. Conversely, in our work, the IGF2BP3 RT-qPCR results significantly correlated with those obtained by IHC, making our results more consistent.

Although both IGF2BP3 mRNA and protein significantly correlated, this correlation was moderate (r = 0.51, *p* < 0.001 and r = 0.46, *p* < 0.001 for H-score 1 and 2, respectively), and the IGF2BP3 protein hardly correlated with the clinicopathologic melanoma features. As expected, similar results were obtained with the two antibodies anti-IGF2BP3 used in IHC analysis, with a significant high correlation between both H-scores (r = 0.86, *p* < 0.001). This makes our IHC results more reliable, showing that both antibodies are suitable for IGF2BP3 protein analysis. However, the Abcam antibody (H-score 1) correlated better with some melanoma parameters. Indeed, IGF2BP3 protein (H-score 1) expression only correlated with ulceration, distant metastasis, and MSS, but (H-score 2) did not show any significant correlation with any melanoma feature. Conversely, IGF2BP3 mRNA expression showed more significant associations with more melanoma parameters and with a higher level of significance. In the present work, RT-qPCR was the most sensitive methodology in detecting more significant relations with the main prognostic clinical and histopathological features, metastasis development, and the poorest survival outcomes. RT-qPCR has a series of widely acknowledged methodological advantages over IHC. It is quantitative by nature, with a much wider dynamic range, quick enough to allow for the rapid therapeutic management of patients, and more sensitive and specific than IHC. Additionally, it does not require an experienced eye, and the results are not affected by subjective interpretations [41,42]. Methodological variations, such as post- transcriptional and translational modifications or the increased dynamic range of RT-qPCR as compared to IHC, could explain the discrepancy observed between mRNA and protein expression. Moreover, mRNA levels are a reflection of the average gene expression in the entire resected tumor, whereas IHC may be biased in favor of selected ‘representative’ tumor areas [41].

Regarding clinical and histopathological variables, the correlation or association of Breslow thickness, the number of mitoses, the presence of ulceration, vertical growth phase, and distant metastasis development with higher levels of IGF2BP3 mRNA obtained in our work is a fact supported by other previous studies that used IHC exclusively. Researchers have reported that IGF2BP3 protein expression is correlated with thick and high-grade tumors and predicts poorer overall, melanoma-specific, recurrence-free, and distant metastasis-free survivals, especially in acral lentiginous melanoma [11,12,13], generating great expectations for its potential prognostic value. Moreover, high levels of IGF2BP3 played an important role in cell proliferation, as well as in the processes of invasion, migration, and metastasis in melanoma [11,15]. IGF2BP3 expression has also been considered for its potential use in the differential diagnosis of malignant melanoma with other benign skin lesions [14,23,24,43]. Although, in the available literature, IGF2BP3 was mostly studied in acral lentiginous melanomas. In our study and in most western countries, the main melanoma subtype was the superficial spreading melanoma (74.3%). Due to the low number of tumors included in the remaining three subtypes, we decided to treat all tumors in the same group for statistical calculations. Nevertheless, our study points to promising results, providing proof that IGF2BP3 would be a reliable prognostic factor even for the earliest stages of melanoma development.

In the Kaplan–Meier analysis, we observed a significant inverse correlation between the IGF2BP3 expression level and the melanoma-specific and distant metastasis-free survival rates. IGF2BP3 expression values over its median showed the poorest MSS and DMFS rates. Multivariate Cox analysis revealed that IGF2BP3 mRNA is a risk factor, independent of Breslow thickness and ulceration, associated with worse survival for both DMFS and MSS. The cut-off values applied in the survival analyses are the medians of IGF2BP3 expression, which are widely used to dichotomize study populations [44,45,46]. Our results indicate that the IGF2BP3 mRNA median value was a valid cut-off which reliably distinguishes between patients with higher and lower risk of developing distant metastasis and dying of melanoma. We also found sequential upregulation of intratumoral expression of IGF2BP3 mRNA over the full spectrum of melanoma progression. Although patients who developed distant metastasis showed a significantly higher expression level of IGF2BP3 mRNA in their primary melanomas, it was not a predictive factor for distant metastasis onset, as indicated by the logistic regression analysis. Therefore, higher levels of IGF2BP3 do not guarantee that the patient will develop distant metastasis at the endpoint. However, it predicts a higher risk of suffering from distant metastasis along the time according to the Cox regression analysis results. Patients with higher IGF2BP3 mRNA levels are at greater risk of earlier distant metastasis development than those with lower levels, and distant metastasis is one of the strongest predictors of shortened survival. Therefore, quantification methods of key melanoma markers that can efficiently predict patients developing metastasis from primary resected tumors may serve as tools for individualizing treatment and improving long-term outcomes. Our data support IGF2BP3 mRNA quantification as a reliable prognostic marker for MSS and DMFS, as previously reported for other types of cancers [17].

In a previous work, our group also evaluated the prognostic value of the intratumoral expression of ten miRNAs implicated in melanoma cell migration and/or invasion in primary tumors for its ability to predict distant metastasis and melanoma survival [3]. The results showed that the downregulation of four of them, has-miR-125b-5p, has-miR-182-5p, has-miR-200c-3p, and has-miR-205-5p, was associated with distant metastatic dissemination and a shorter survival. Indeed, three of them, has-miR-125b-5p, has-miR-205-5p, and has-miR-200c-3p, were independent predictors of MSS [3]. In addition, in the present work, we revealed that the downregulation of these four miRNAs also correlated with IGF2BP3 mRNA expression, in agreement with the results obtained in the survival analysis for IGF2BP3, which turned out to also be a prognostic factor for both DMFS and MSS, independently of Breslow thickness and ulceration.

## 5. Conclusions

In conclusion, high IGF2BP3 mRNA levels are associated with a more aggressive tumor behavior and worse clinical outcomes, supporting its use as an effective prognostic biomarker even in the earlier stages of melanoma development. RT-qPCR assessment of IGF2BP3 discriminated more effectively than IHC and predicted better clinical outcomes. The quantification of IGF2BP3 mRNA expression with RT-qPCR represents a promising alternative to conventional visual estimation by IHC and may assist in improving reproducibility and accuracy in the field. Our findings highlight the feasibility of using RT-qPCR, instead of IHC, for the routine assessment of IGF2BP3 in order to assist in the selection of melanoma patients with a higher risk of developing distant metastasis or dying from melanoma. Due to the relatively small sample size, these data should be validated in larger datasets.

## Figures and Tables

**Table 1:** Primary melanomas histological and clinical characteristics.

Primary Melanomas (*n* = 70)	
Variable	Number of Cases (%)
Breslow Thickness (mm)	
≤1	32 (45.7)
1.01–2.00	14 (20.0)
2.01–4.00	8 (11.4)
>4	16 (22.9)
Ulceration	
Absent	53 (75.7)
Present	17 (24.3)
Mitosis/mm^2^	
0	23 (32.9)
≥1	47 (67.1)
Growth phase	
Radial	22 (31.4)
Vertical	48 (68.6)
Location	
Limbs	28 (40.0)
Trunk	35 (50.0)
Head and neck	7 (10.0)
Gender	
Female	46 (65.7)
Male	24 (34.3)
Histological type *	
SSM	52 (74.29)
LMM	5 (7.14)
ALM	5 (7.14)
NM	8 (11.43)
Age at diagnosis (years)	
≤65	38 (54.3)
>65	32 (45.7)
In-Transit Metastasis	
Absent	61 (87.1)
Present	9 (12.9)
Lymph Node Metastasis	
Absent	56 (80.0)
Present	14 (20.0)
Distant Metastasis	
Absent	51 (72.9)
Present	19 (27.1)
Any Metastasis	
Absent	48 (68.6)
Present	22 (31.4)
Melanoma-specific Survival	
Alive	55 (78.6)
Dead	15 (21.4)
Follow-up (months)	
Mean, Range	96.1 (6.6–210.4)

* Superficial Spreading Melanoma (SSM), Lentigo Maligna Melanoma (LMM), Acral lentiginous Melanoma (ALM), and Nodular Melanoma (NM).

**Table 2:** List of TaqMan^®^MicroRNA assays used in this study.

Assay Name	Assay ID	Mature miRNA Sequence
hsa-miR-9–5p	000583	UCUUUGGUUAUCUAGCUGUAUGA
hsa-miR-21-5p	000397	UAGCUUAUCAGACUGAUGUUGA
hsa-miR-125b-5p	000449	UCCCUGAGACCCUAACUUGUGA
hsa-miR-137	001129	UUAUUGCUUAAGAAUACGCGUAG
hsa-miR-182-5p	002334	UUUGGCAAUGGUAGAACUCACACU
hsa-miR-191-5p	002299	CAACGGAAUCCCAAAAGCAGCUG
hsa-miR-200c-3p	002300	UAAUACUGCCGGGUAAUGAUGGA
hsa-miR-205-5p	000509	UCCUUCAUUCCACCGGAGUCUG
hsa-miR-211-5p	000514	UUCCCUUUGUCAUCCUUCGCCU
hsa-miR-221-3p	000524	AGCUACAUUGUCUGCUGGGUUUC
RNU48	001006	GAUGACCCCAGGUAACUCUGAGUGUGUCG
		CUGAUGCCAUCACCGCAGCGCUCUGACC

**Table 3:** Relation between IGF2BP3 (mRNA and protein) expression and clinicopathologic prognostic factors.

	IGF2BP3 mRNA (*n* = 61)			IGF2BP3 Protein (1) (*n* = 63)			IGF2BP3 Protein (2) (*n* = 63)			
Variable	IGF2BP3 mRNA (2^-∆Ct^)	*p*-Value	Correlation Coefficient	IGF2BP3 Protein (H-Score 1)	*p*-Value	Correlation Coefficient	IGF2BP3 Protein (H-Score 2)	*p*-Value	Correlation Coefficient	Test
IGF2BP3 median value	0.654	-	-	40	-	-	22.90	-	-	-
Breslow thickness (mm)										
Range (0–20)	(0.039–251.701)	<0.001 ***	0.54	(0.00–163.33)	0.06	0.24	(0.00–190.00)	0.16	0.18	Spearman
Breslow thickness categories (mm)										
≤1 (T1)	0.344	<0.001 ***	-	31.66	0.379		6.60	0.772	-	Kruskal-Wallis
1.01–2.00 (T2)	0.514			30.00			29.00			
2.01–4.00 (T3)	0.763			2.66			2.5			
>4 (T4)	3.426			61.66			23.30			
Mitosis/mm^2^										
Range (0–27)	(0.039–251.701)	<0.001 ***	0.43	(0.00–163.33)	0.06	0.24	(0.00–190.00)	0.23	0.15	Spearman
Ulceration										
Absent	0.453	<0.001 ***	-	30.00	0.037 *	-	16.65	0.129	-	Wilcoxon
Present	3.220			62.08			32.50			
Growth phase										
Radial	0.471	0.032 *	-	30.00	0.225	-	15.80	0.63	-	Wilcoxon
Vertical	1.367			40.63			22.90			
Metastasis										
In-transit metastasis										
Absent	0.558	0.132	-	40.00	0.268	-	25.00	0.155	-	Wilcoxon
Present	1.999			7.50			2.50			
Lymph node metastasis										
Absent	0.471	<0.001 ***	-	31.66	0.129	-	3.30	0.944	-	Wilcoxon
Present	2.291			62.50			22.50			
Distant metastasis										
Absent	0.433	<0.001 ***	-	30.00	0.031 *	-	19.15	0.372	-	Wilcoxon
Present	2.354			66.25			26.25			
Any metastasis										
Absent	0.393	<0.001 ***	-	30.00	0.135	+	23.30	0.625	-	Wilcoxon
Present	2.388			61.66			22.50			
Melanoma-specific survival										
Alive	0.534	0.009 **	-	30.00	0.029 *		16.65	0.2336	-	Wilcoxon
Dead	2.291			83.33			32.50			

*** Significant at *p* < 0.001, ** Significant at *p* < 0.01, and * Significant at *p* < 0.05. (1) refers to the IHC results obtained using the anti-IGF2BP3 antibody from Abcam (H-score 1), and (2) refers to those obtained using the anti-IGF2BP3 antibody from Dako (H-score 2). For continuous variables such as Breslow thickness and number of mitoses, IGF2BP3 mRNA and protein values are expressed as a range, and correlation was analyzed between each variable and IGF2BP3 (mRNA or protein) expression. For categorical variables with two or more groups (Breslow thickness categories, presence or absence of ulceration, growth phase, metastasis development, and melanoma-specific survival), IGF2BP3 values (mRNA or protein) are expressed as the median in each group, and comparisons among groups were performed.

**Table 4:** Multivariate and univariate Cox regression analysis for DMFS and MSS.

	**DMFS**	**Multivariate**			**Univariate**		
		**HR (Exp(B))**	**95% CI**	***p*** **-Value**	**HR (Exp(B))**	**95% CI**	***p*** **-Value**
IGF2BP3 mRNA	Breslow	1.307	1.166–1.466	<0.001 ***	1.315	1.198–1.443	<0.001 ***
	Ulceration	3.423	1.149–10.194	0.027 *	10.360	3.773–28.430	<0.001 ***
	IGF2BP3 mRNA	1.016	1.005–1.028	0.004 **	1.015	1.006–1.024	0.001 **
IGF2BP3 protein (H-score 1)	Breslow	1.299	1.158–1.459	<0.001 ***	1.332	1.213–1.462	<0.001 ***
	Ulceration	4.785	1.563–14.643	0.006 **	11.020	4.010–30.310	<0.001 ***
	IGF2BP3 protein	1.008	0.999–1.015	0.053	1.005	0.999–1.011	0.078
IGF2BP3 protein (H-score 2)	Breslow	1.297	1.159–1.458	<0.001 ***	1.332	1.213–1.462	<0.001 ***
	Ulceration	4.061	1.390–11.862	0.104 *	11.020	4.010–30.310	<0.001 ***
	IGF2BP3 protein	1.002	0.993–1.010	0.651	1.003	0.9944–1.012	0.487
	**MSS**	**Multivariate**			**Univariate**		
		**HR (Exp(B))**	**95% CI**	***p*** **-Value**	**HR (Exp(B))**	**95% CI**	***p*** **-Value**
IGF2BP3 mRNA	Breslow	1.143	1.024–1.275	0.017 *	1.171	1.077–1.273	<0.001 ***
	Ulceration	3.675	1.159–11.647	0.027 *	7.379	2.439–22.320	<0.001 ***
	IGF2BP3 mRNA	1.024	1.100–1.048	0.044 *	1.066	1.008–1.127	0.0243 *
IGF2BP3 protein (H-score 1)	Breslow	1.123	0.999–1.263	0.051	1.175	1.085–1.237	<0.001 ***
	Ulceration	5.396	1.644–17.715	0.005 **	7.840	2.590–23.730	<0.001 ***
	IGF2BP3 protein	1.004	0.996–1.013	0.322	1.005	0.999–1.012	0.091
IGF2BP3 protein (H-score 2)	Breslow	1.146	1.024–1.282	0.017 *	1.175	1.085–1.273	<0.001 ***
	Ulceration	4.689	1.498–14.674	0.008 **	7.840	2.590–23.730	<0.001 ***
	IGF2BP3 protein	1.002	0.993–1.011	0.678	1.005	0.9962–1.014	0.258

* *p* < 0.05, ** *p* < 0.01, and *** *p* < 0.001.

**Table 5:** Multivariate logistic regression analysis for distant metastasis occurrence.

	Coefficients	Estimate	Std. Error	z Value	*p*-Value
IGF2BP3 mRNA	(Intercept)	−2.951	0.645	−4.547	<0.001 ***
	Breslow	0.435	0.171	2.550	0.011 *
	Ulceration	1.507	0.973	1.549	0.121
	IGF2BP3 mRNA	0.013	0.019	0.698	0.485
IGF2BP3 protein (H-score 1)	(Intercept)	−3.453	0.839	−4.118	<0.001 ***
	Breslow	0.720	0.294	2.449	0.014 *
	Ulceration	0.494	1.272	0.388	0.698
	IGF2BP3 protein	0.005	0.007	0.663	0.508
IGF2BP3 protein (H-score 2)	(Intercept)	−2.975	0.750	−3.967	<0.001 ***
	Breslow	0.698	0.289	2.420	0.016 *
	Ulceration	0.785	1.294	0.607	0.544
	IGF2BP3 protein	−0.007	0.011	−0.606	0.545

* *p* < 0.05 and *** *p* < 0.001.

## Data Availability

The data presented in this study are available from the corresponding author upon reasonable request.

## Supplementary Materials

The following supporting information can be downloaded at: https://www.mdpi.com/article/10.3390/cancers14092319/s1. Figure S1: Analysis of has-miR-125b-5p, has-miR-182-5p, has-miR-200c-3p, has-miR-205-5p, and has-miR-9-5p in primary human melanoma tissue samples in relation to IGF2BP3 mRNA and protein expression (H-scores 1 and 2), Figure S2: Association of has-miR-125b-5p, has-miR-182-5p, has-miR-200c-3p, has-miR-205-5p, and has-miR-9-5p according to IGF2BP3 mRNA and protein median values (H-scores 1 and 2), Table S1: Relation between IGF2BP3 (mRNA and protein) expression and miRNA expression, and Table S2: Raw data (Ct mean from the triplicates) of IGF2BP3 mRNA quantification by RT-qPCR.
