# Supplementary material for: Prognostic Value of IGF2 mRNA-Binding Protein 3 (IGF2BP3) Intratumoral Expression in Melanoma Patients at the Time of Diagnosis: Comparative Analysis of RT-qPCR Versus Immunohistochemistry
Source: Cancers (Basel). 2022 May 7;14(9):2319. doi: 10.3390/cancers14092319 (PMC9100051; doi:10.3390/cancers14092319)
Supplement: Supplementary file 1 [file cancers-14-02319-s001.zip › cancers-1711961-supplementary.pdf]

cancers

MDPI

Supplementary File

Prognostic value of IGF2 mRNA-binding protein 3 (IGF2BP3) intratumoral expression in melanoma patients at the time of diagnosis: comparative analysis of RT-qPCR versus immunohistochemistry

Beatriz Sánchez-Sendra, Silvia Pérez-Debén, José F. González-Muñoz, Amelia Murgui and Carlos Monteagudo

hsa-miR-125b-5p, hsa-miR-182-5p, hsa-miR-200c-3p, hsa-miR-205-5p and hsa-miR-9-5p expression correlate with IGF2BP3 in primary melanoma tumors

We also analyzed the correlation between IGF2BP3 (mRNA and protein) expression and the expression of ten miRNAs previously described to be implicated in melanoma pathogenesis [3,5,8,9] (subject of study in our previous work (Sánchez-Sendra B 2018)). The importance of the correlation analysis between the studied miRNAs and IGF2BP3 (mRNA and protein) resides in the fact that they are all (miRNAs and IGF2BP3) prognostic markers in melanoma. Among miRNAs, hsa-miR-125b-5p, hsa-miR-182-5p, hsa-miR-200c-3p and hsa-miR-205-5p showed a significant inverse Spearman correlation with IGF2BP3 mRNA (p = 0.003, r = –0.378; p = 0.004, r = -0.365; p < 0.001, r = –0.469 and p < 0.001, r = -0.549, respectively) and hsa-miR-9-5p correlated positively and significantly with IGF2BP3 protein H-score 1 (p = 0.023, r = 0.311) (Figure S1a, b) (Table S1). However, no significant correlation was observed between IGF2BP3 protein H-score 2 and any of the ten miRNAs studied (Figure S1c) (Table S1). We also compared the expression of each miRNA according to the median value of IGF2BP3 (mRNA or protein) expression (Figure S2a, b, c). hsa-miR-200c-3p and hsa-miR-205-5p, exhibited a significant lower expression when IGF2BP3 mRNA expression was higher than its median value (p = 0.008 and p = 0.001, respectively) (Figure S2a). Conversely, hsa-miR-9-5p expression was significantly higher for IGF2BP3 mRNA values over its median (p = 0.023) (Figure S2a). There were not significant differences when miRNAs expressions were compared according to IGF2BP3 protein 1 and protein 2 medians (Figure S2b, c).

Table S1. Relation between IGF2BP3 (mRNA and protein) expression and miRNA expression.

	**IGF2BP3 mRNA (*n* = 61)**			**IGF2BP3 protein (1) (*n* = 63)**			**IGF2BP3 protein (2) (*n* = 63)**		
**Variable**	**IGF2BP3 mRNA (2-ΔCt)**	**p-value**	**Spearman correlation coefficient**	**IGF2BP3 protein (H-score 1)**	**p-value**	**Spearman correlation coefficient**	**IGF2BP3 protein (H-score 2)**	**p-value**	**Spearman correlation coefficient**
				**miRNAs (2-ΔCt)[a]**					
hsa-miR-125b-5p		0.003**	-0.378		0.177	-0.188		0.180	-0.187
hsa-miR-137		0.339	0.125		0.394	0.119		0.569	0.080
hsa-miR-182-5p		0.004**	-0.365		0.989	0.002		0.947	0.009
hsa-miR-191-5p		0.640	0.061		0.429	0.111		0.731	0.048
hsa-miR-200c-3p	(0.039 - 251.701)	< 0.001***	-0.469	(0.00 - 163.33)	0.206	-0.177	(0.00 -190.00)	0.244	-0.163
hsa-miR-205-5p		< 0.001***	-0.549		0.096	-0.231		0.069	-0.252
hsa-miR-21-5p		0.801	0.033		0.938	0.011		0.696	-0.055
hsa-miR-211-5p		0.251	-0.149		0.582	0.077		0.533	0.088
hsa-miR-221-3p		0.747	0.042		0.724	0.050		0.720	0.050
hsa-miR-9-5p		0.055	0.247		0.023*	0.311		0.088	0.237

***Significant at p <0.001, **Significant at p <0.01, *Significant at p <0.05. [a] miRNAs described in literature to be implicated in melanoma cell migration and/or invasion. Some of them have been proposed as prognostic biomarkers of high-risk melanoma patients. For the continuous miRNA expression variable, IGF2BP3 mRNA and protein (H-score 1 and 2) values are expressed as a range and the correlation was analyzed between miRNA expression and both, IGF2BP3 mRNA and protein (H-score 1 and 2) expression.

Table S2. Raw data (Ct mean from the triplicates) of IGF2BP3 mRNA quantification by RT-qPCR.

Sample code	IGF2BP3 Ct Mean	18S Ct Mean	Sample code	IGF2BP3 Ct Mean	18S Ct Mean	Sample code	IGF2BP3 Ct Mean	18S Ct Mean
1	29.77	10.57	22	33.45	10.88	43	34.04	12.43
2	35.89	13.78	23	31.59	14.54	44	33.30	11.92
3	31.31	13.42	24	27.29	15.33	45	36.43	15.36
4	35.78	12.70	25	29.39	10.67	46	30.53	11.70
5	35.53	14.39	26	32.90	16.07	47	33.71	16.19
6	36.26	15.80	27	38.16	15.14	48	32.68	15.42
7	37.43	14.33	28	38.55	13.93	49	33.97	14.73
8	32.11	13.94	29	34.09	14.98	50	34.69	11.74
9	26.85	11.26	30	30.16	13.64	51	33.01	11.44
10	34.51	15.83	31	36.67	15.65	52	34.70	13.86
11	35.40	15.64	32	32.85	11.45	53	29.39	12.35
12	37.93	13.64	33	31.01	12.67	54	35.65	13.22
13	36.55	14.58	34	33.83	13.05	55	32.96	12.41
14	37.49	15.47	35	35.23	15.52	56	33.53	11.27
15	32.22	11.92	36	36.38	13.98	57	34.01	13.88
16	38.97	15.32	37	34.10	11.33	58	30.88	14.63
17	30.50	11.51	38	30.25	12.10	59	28.53	13.80
18	33.69	12.50	39	35.63	12.04	60	33.98	13.22
19	34.73	15.90	40	29.45	13.65	61	31.89	10.76
20	34.33	13.54	41	37.01	14.10	qPCR negative controls	Undetermined	Undetermined
21	33.62	13.60	42	30.68	11.92	RT negative controls	Undetermined	Undetermined

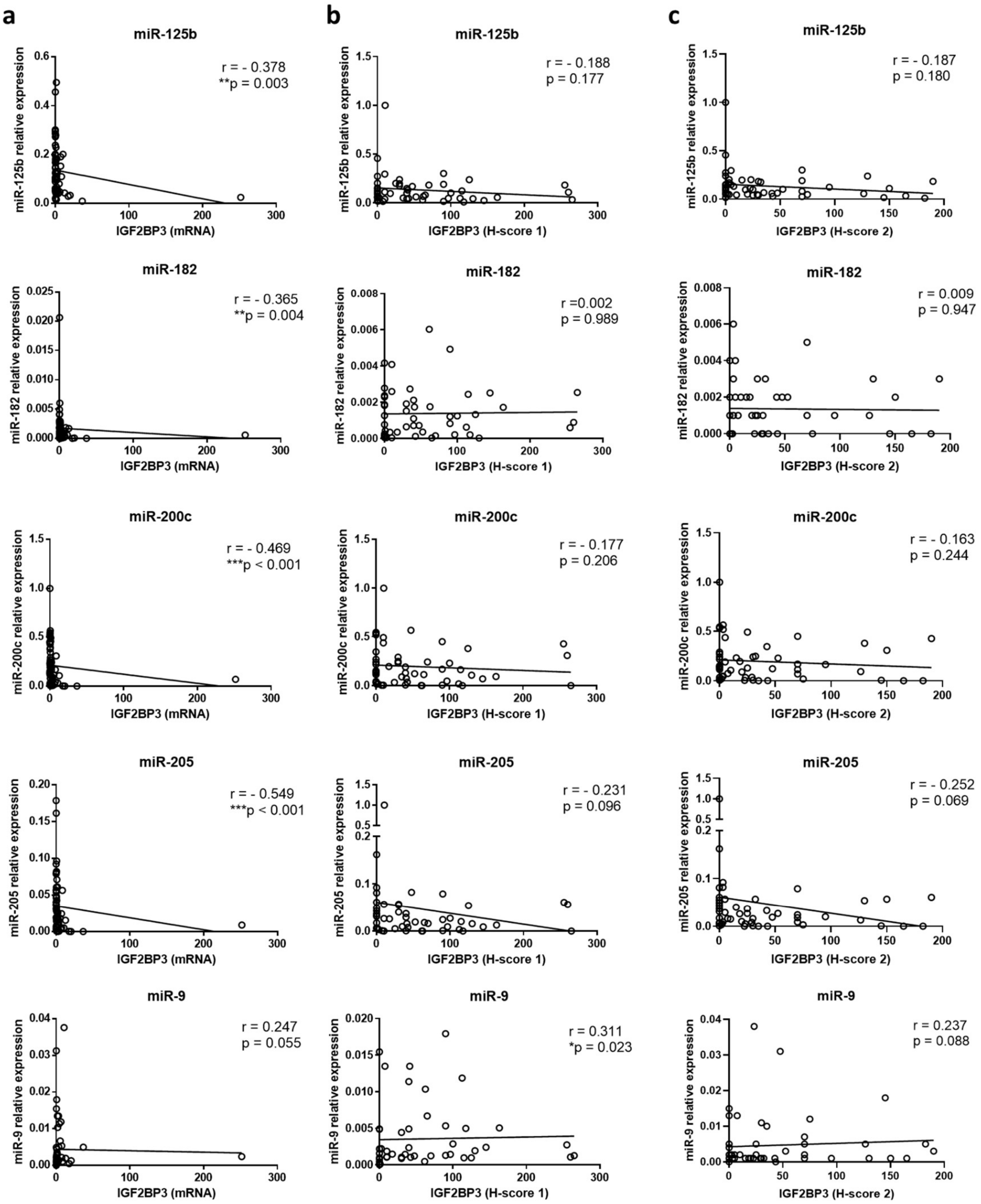

Figure S1. Analysis of hsa-miR-125b-5p, hsa-miR-182-5p, hsa-miR-200c-3p, hsa-miR-205-5p and hsa-miR-9-5p in primary human melanoma tissue samples in relation to IGF2BP3 mRNA and protein expression (H-scores 1 and 2). Correlation of relative expression of hsa-miR-125b-5p, hsa-miR-182-5p, hsa-miR-200c-3p, hsa-miR-205-5p and hsa-miR-9-5p with (**a**) IGF2BP3 mRNA expression, and with IGF2BP3 protein levels (**b**) H-score 1 and (**c)** H-score 2. *p<0.05, **p<0.01 and ***p<0.001.

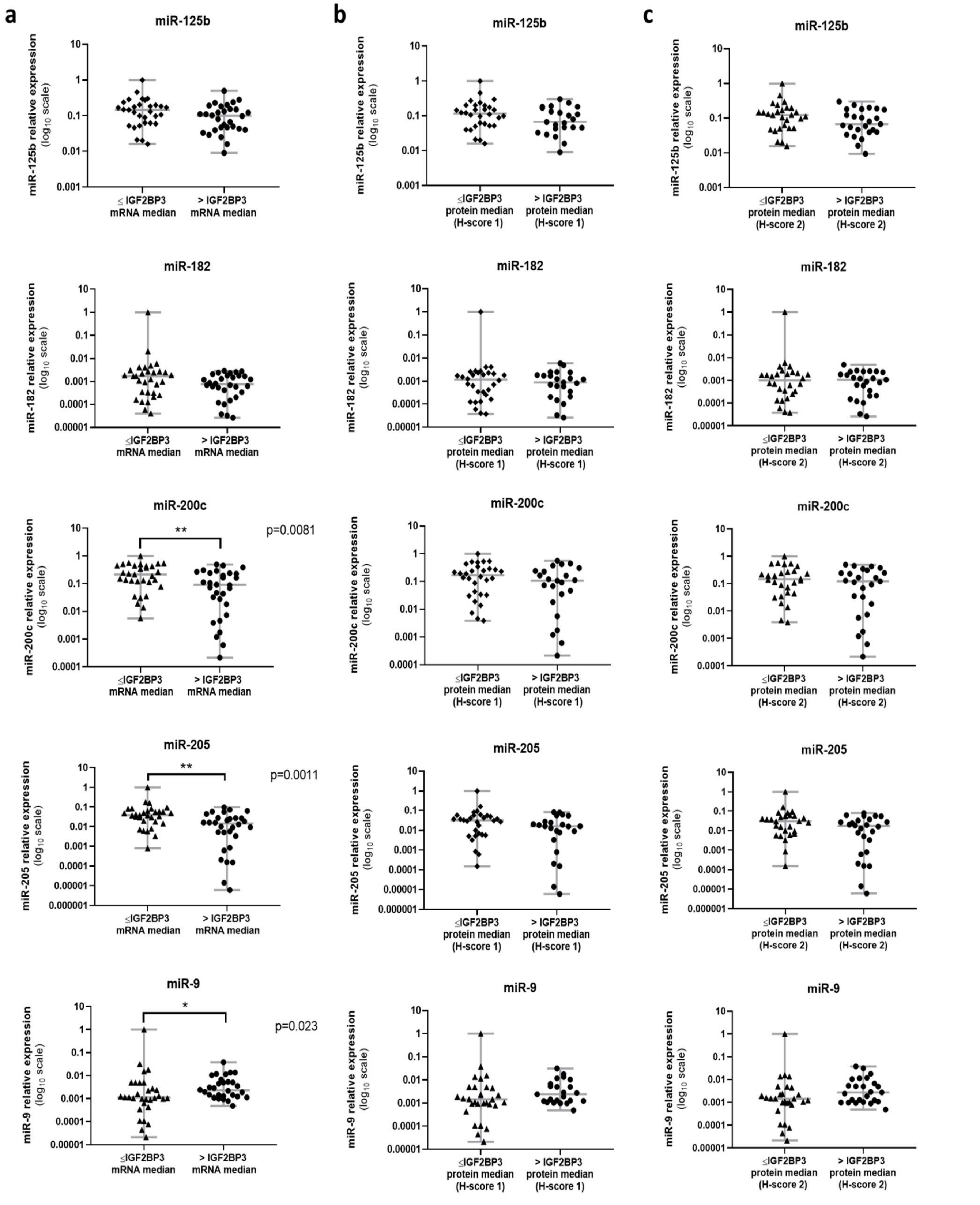

Figure S2. Association of hsa-miR-125b-5p, hsa-miR-182-5p, hsa-miR-200c-3p, hsa-miR-205-5p and hsa-miR-9-5p according to IGF2BP3 mRNA and protein median values (H-scores 1 and 2). Analysis of hsa-miR-125b-5p, hsa-miR-182-5p, hsa-miR-200c-3p, hsa-miR-205-5p and hsa-miR-9-5p expression with (**a**) IGF2BP3 mRNA (**b**) protein H-score 1 and (**c**) H-score 2 median values. *p<0.05, **p<0.01.
